# Factors associated with inadequate dietary diversity among adolescent girls in Hurumu Woreda High School, Oromia Region, Southwest Ethiopia

**DOI:** 10.3389/fnut.2024.1234224

**Published:** 2024-02-09

**Authors:** Abeza Mitiku Kera, Asrat Zewdie Zenebe, Keno Melkamu Kitila, Zewudu Befkadu Tola, Teshome Bekana

**Affiliations:** ^1^Department of Public Health, College of Health Science, Mattu University, Mattu, Ethiopia; ^2^Department of Biomedical Science, College of Health Science, Mattu University, Mattu, Ethiopia; ^3^Department of Medical Laboratory Science, College of Health Science, Mattu University, Mattu, Ethiopia

**Keywords:** dietary diversity, adolescent girls, nutrition, Ethiopia, Hurumu Woreda

## Abstract

**Background:**

Inadequate diet among adolescent girls leads to anatomical and physiological disturbances which will contribute to the vicious intergenerational cycle of malnutrition. However, only a few studies are available in Ethiopia on dietary diversity among adolescent girls who are attending school.

**Objective:**

The objective of this study is to assess factors associated with inadequate dietary diversity among high school adolescent girls in Hurumu Woreda, Southwest Ethiopia, 2022.

**Methods:**

An institution-based cross-sectional study was conducted among 374 high school adolescent girls from 3 May 2022 to 12 June 2022 and selected by using simple random sampling techniques. Data were collected through face-to-face interviews using structured questionnaires. Epi-data version 4.6.0 was used to enter the data, which were then exported to SPSS version 26 for analysis. Simple binary and multivariable logistic regressions were performed to identify factors associated with inadequate dietary diversity.

**Results:**

In this study, the magnitude of inadequate dietary diversity among adolescent girls was 62.6% [95% CI: 57.5–66.5]. Living with more than five family members (AOR = 1.8, 95% CI: 1.16–3.44), consumption of sweet foods/beverages (AOR = 2.2, 95% CI: 1.07–3.41), poor nutritional knowledge (AOR = 2.5, 95% CI: 1.48–3.89), and poor household wealth tercile (AOR = 2.8, 95% CI: 1.44–5.12) were significantly associated with inadequate dietary diversity.

**Conclusion:**

Living with more than five family members, poor household wealth status, consumption of sweet foods/beverages, family size, and poor nutritional knowledge were factors significantly associated with inadequate dietary diversity. Hence, nutrition education, the use of family planning methods, and securing income-generating activities should be implemented.

## 1 Background

Adolescence is a period of growth and development in a human’s life that necessitates proper nutrition to ensure healthy growth, development, sexual maturation, and future health. It is a window of opportunity for optimal growth and development in order to prevent or reduce the risk of non-communicable diseases (1). It is one of the few opportunities for adolescent girls to break the vicious cycle of malnutrition transmission, in which an undernourished girl is more likely to have nutritionally impaired children (2, 3).

Dietary diversity is defined as basic count of food types ingested throughout specified reference period. Nutritional status and nutrient adequacy can be accurately predicted by an individual’s dietary diversity score (4). In practice, dietary diversity can be measured at an individual or household level, and the measurement of dietary diversity at the household level serves as surrogate for family food security or insecurity (4, 5).

Globally, adolescent diets are characterized by insufficiently diverse food, less nutrient-dense food, and more processed foods and beverages, resulting in the emergence of various micronutrient deficiencies such as, vitamin A, iron, and iodine (6–8). Due to the predominance of a high proportion of starchy staples in all diets in low-income and middle-income countries, inadequate dietary diversity is more prevalent across all age groups (9–11), and similarly, in Ethiopia, studies conducted in Jimma town, Addis Ababa, Woldia, and Wolaita Sodo revealed that 61.3, 43.3, 49.1, and 72.4% of school adolescent girls had inadequate dietary diversity, respectively (8, 12–14).

Inadequate diet among adolescent girls ended up causing anatomical and physiological disturbances, poor academic achievement, poor physical and reproductive maturity, and other macronutrient and micronutrient deficiencies which have a negative impact on their present and future health (1, 9, 15). Household economic status, uneven intra-familial food distribution, particular food taboos, dietary restrictions during menstruation, peer pressure, food choices, culture, mass media, and body image perception could all influence adolescents’ dietary consumption and food selection (12, 16, 17).

According to studies conducted in different regions of Ethiopia, several factors such as nutritional knowledge, residence, wealth status, maternal education, maternal occupation, and family size were identified as factors associated with the dietary diversity of adolescent girls (8, 12–14).

Gender equality and good nutrition for women and adolescent girls are prerequisites for achieving all 17 Sustainable Development Goals (SDG), and it is one of the core activities for achieving the UN Agenda 2030 (1, 4). The Ethiopian Government has tried various strategies, such as school feeding programs, to intervene in adolescent nutrition. However, the school feeding program is limited and cannot reach Ethiopian communities in marginalized areas (17).

Adolescent girls face multiple challenges in getting a diversified diet. Uneven household food division is one of the challenges facing adolescent girls in Ethiopia (17). However, there are few studies on adolescent girls’ dietary diversity in developing countries, including Ethiopia, and previously, there was no similar study conducted in Hurumu Woreda. Hence, this study was designed to assess factors associated with inadequate dietary diversity among high school adolescent girls in Hurumu Woreda, which will provide a direction for future strategic planning.

## 2 Materials and methods

### 2.1 Study area and period

The study was carried out in Hurumu Woreda High School from 3 May 2022 to 12 June 2022. Hurumu Woreda is located in the Ilubabor zone of Oromia regional, 584 kilometers from Ethiopia’s capital city, Addis Ababa. In the woreda, there are two high schools. The area is best known for its coffee cultivation. There were 2,508 students enrolled in the two high schools, with 1,276 of them being adolescent girls.

### 2.2 Study design and population

An institutional-based cross-sectional study design was employed from 3 May 2022 to 12 June 2022. All adolescent girls attending school in the 2021/2022 academic year were the source population, whereas randomly selected adolescent girls from the two high schools were the study population and included in this study.

### 2.3 Eligibility criteria

#### 2.3.1 Inclusion criteria

Adolescent girls found in the 14–19 year age range and who were attending high school (grades 9–12).

#### 2.3.2 Exclusion criteria

Adolescent girls who were fasting, either for religious reasons or to maintain their shape, those who had a ceremony 1 day before the data collection, and those who were currently recovering from acute illness were excluded.

### 2.4 Sample size determination

The required sample size was calculated using a single population proportion formula by taking a proportion of inadequate dietary diversity as 61.3% (12), a level of confidence of 95%, and a margin of error of 5%. The initial sample size was 365. By considering a 5% non-response rate, the final sample size was 383.

### 2.5 Sampling techniques and procedures

In the woreda, there are two high schools. There were 1,276 adolescent girls enrolled in the two high schools during the study period. The list of adolescent girls was then compiled by the respective school directors from each school and combined into a single data set. In the data set, information such as name, age, and grade level was included since we used it as a sampling frame. The required number of adolescent girls who will be selected from each high school was determined based on a proportional population size allocation. Finally, through a simple random sampling technique, 383 students were selected using the open epi computer generator random number.

### 2.6 Data collection procedures and tools

Data were collected using a structured interviewer-administered questionnaire. The questionnaire was adapted from previous similar studies and guidelines (8, 12, 13, 18–22). The questionnaire was divided into four sections: sociodemographic and economic information; adolescent meal patterns and related behaviors; nutritional knowledge; and dietary diversity score, which was adopted from FAO (4). It was composed of 10 food groups (cereals, pulses, nuts and seeds, milk and milk products, meats and fish, eggs, dark green leafy vegetables, other vitamin A-rich fruits and vegetables, other vegetables, and other fruits). The questionnaire was initially prepared in English and subsequently translated into Afan Oromo and back into English to verify its consistency by an expert who was proficient in both languages. Participants were asked to recall all foods and beverages they consumed within the last 24 h preceding the survey.

Data were collected by four diploma nurses, and two BSc nurses were recruited as supervisors during data collection. Data were gathered through a school-to-school visit of adolescent girls in their classes. To complete the interview, it took 20–30 min. If eligible students were absent during the data collection period, a revisit of their class was done three times, and adolescent girls absent on the third visit were considered non-respondents.

### 2.7 Data quality control

Before data collection, the questionnaires were pretested on a 5% sample size in another high school, which was not a part of the actual data collection area. Based on the pretest, some modifications, such as unclear or vague questions and wrong skip patterns, were corrected. Overall, 2 days of training were given to data collectors and supervisors by the principal investigator on data collection tools, data collection techniques, the approach to the interviews, and maintaining the privacy and confidentiality of the respondents. Every day after data collection, the questionnaires were reviewed and checked for completeness by the supervisors and the principal investigator. Questionnaires were reviewed and checked for completeness, word errors, unclear questions, and consistency by the supervisor and principal investigator, and the necessary feedback was given to data collectors each morning.

**Adolescent girls** are girls ranging in age from 10 to 19 years. Early adolescence (ages 10–13 years), middle adolescence (ages 14–16 years), and late adolescence (17–19 years) (1).

**Dietary diversity score:** Adolescent girls who consumed less than five food groups out of ten (10) were classified as having inadequate dietary diversity, while those who consumed five or more food groups were classified as having adequate dietary diversity. It was coded as “1” for inadequate dietary diversity and “0” for adequate dietary diversity (13).

**Ceremony**: a formal religious or public event held on special occasions such as weddings, birthday, and graduations.

**Eating outside**: eating meals from outdoor sources such as restaurants and cafeteria.

**Wealth index**: It is measured using fixed household assets. The factor score was derived using principal component analysis (PCA), and then, the composite score was ranked into three tertiles (19).

**Adolescent nutritional knowledge**: Respondents whose knowledge scores were equal to or above the mean were categorized as having good knowledge, whereas respondents whose knowledge scores were below the mean were categorized as having poor nutritional knowledge (14).

### 2.8 Data processing and analysis

All data were checked visually, coded, and entered into Epi-data version 4.6.0 before being exported to SPSS version 26 for analysis. Descriptive statistics (frequency and cross-tabulation) were calculated for variables. The results were presented in the form of tables and text using frequencies and summary statistics such as mean, standard deviation, and percentage, to describe the study population with relevant variables.

The degree of association between independent and dependent variables was assessed using an odds ratio with a 95% confidence interval. A simple binary logistic regression analysis was performed to select candidate variables for multivariable analysis. Variables with a *p*-value < 0.25 were taken as a cutoff point to select eligible variables for the multiple regression analysis, and a *p*-value < 0.05 was declared statistically significant in the final model.

Pseudo-regression was performed to check multi-collinearity between independent variables; the minimum tolerance and maximum variance inflation (VIF) factors were found to be 0.63 and 1.81, respectively. For the finally fitted multivariable logistic regression model, the adequacy of the model to predict the outcome variables was checked by Hosmer–Lemeshow goodness-of-fit and *p*-value > 0.05 (0.46).

## 3 Results

### 3.1 Socioeconomic and demographic characteristics of participants

A total of 374 school adolescent girls participated in the study, making a response rate of 97.6%. The mean age of the respondents was (16.6 SD ± 1.24) years which was in the age range of 17–19 years. Majority of the participants 294 (78.6%) live with more than five family members. In terms of wealth index, approximately one-third 114 (30.5%) of the adolescent live in the household with poor tercile index. Regarding the educational status of parents, 69 (18.4%) of their fathers and 76 (20.3%) of mothers have no formal education. By occupation, 223 (59.6%) of their fathers and 176 (47.1%) of mothers were farmer and housewife, respectively (Table 1).

**Table 1:** Sociodemographic characteristics of school adolescent girls in Hurumu Woreda High Schools, Oromia Region, Southwest Ethiopia, 2022.

Variable (*n* = 374)	Category	Frequency	Percent
Age of students	14–16	110	29.4
	17–19	264	70.6
Grade level	9–10	222	59.4
	11–12	152	40.6
Ethnicity	Oromo	307	82.1
	Amhara	36	9.6
	Tigre	17	4.5
	Other^a^	14	3.7
Religion	Protestant	134	35.8
	Orthodox	128	34.2
	Muslim	112	29.9
Residence	Urban	81	21.7
	Rural	293	78.3
Educational status of the mother	No formal education	76	20.3
	Primary education	151	40.4
	Secondary and above	147	39.3
Occupation of the mother	Housewife	176	47.1
	Merchant	97	25.9
	Government employee	60	16.0
	Other^b^	41	11.0
Educational status of the father	No formal education	69	18.4
	Primary education	121	32.4
	Secondary and above	184	49.2
Occupation of the father	Farmer	223	59.6
	Government employed	67	17.9
	Merchant	52	13.9
	Daily labor	32	8.6
Family size	<5 members	80	21.4
	>5 members	294	78.6
Household decision on food	Father	249	66.6
	Mother	76	20.3
	Both mother and father	49	13.1
Wealth status	Poor	114	30.5
	Medium	122	32.6
	Rich	138	36.9

a Gurage and Kafa.

b Daily laborer and farmer.

### 3.2 Meal pattern and related behavior among school adolescent girls in Hurumu Woreda High Schools

Approximately one-fifth of the participants, 87 (23.3%), have never eaten their meals outside. Moreover, 107 (28.6%) of the participants had consumed sweet food or beverages. Regarding the meal frequency of the participants, more than one-fourth, 84 (22.5%), of them eat two times per day, while 74 (19.8%) of them skip their breakfast (Table 2).

**Table 2:** Meal pattern and related behavior among school adolescent girls in Hurumu Woreda High Schools, Oromia Region, Southwest Ethiopia, 2022.

Variable (*n* = 374)	Category	Frequency	Percent
Eating out side	Never	87	23.3
	At least once per week	287	76.7
Consumption of sweet foods/beverages	Yes	107	28.6
	No	267	71.4
Meal frequency	Two times per day	84	22.5
	3–4 times per day	290	77.5
Eating companions within the family	With family members	196	52.40
	With siblings	149	39.80
	Alone	29	7.80
Did you skip eating your breakfast in the last 24 h	Yes	74	19.8
	No	300	80.2
Reason for skipping breakfast	I do not like to eat early	36	48.7
	No time to have breakfast	18	24.3
	My family skip the breakfast	12	16.2
	I do not like the food choices	8	10.8

### 3.3 Nutritional knowledge of adolescent girls in Hurumu Woreda High School

In terms of participants’ nutritional knowledge, 306 (81.8%) of them responded that a diet containing more fruits and vegetables is good to prevent certain diseases, while 221 (59.2%) and 178 (47.6%) know the dietary sources of carbohydrates and protein, respectively. Of the study participants, 221 (66.0%) were aware of the health consequences of excessive salt consumption. However, only 74 (19.8%) were aware of the dietary sources of fiber. The mean knowledge score of the participants was 5.33 (SD + 1.72). Thus, 211 (56.4%) and 163 (43.6%) of the participants were categorized as having poor and good nutritional knowledge, respectively (Table 3).

**Table 3:** Nutritional knowledge of adolescent girls in Hurumu Woreda High Schools, Oromia Region, Southwest Ethiopia, 2022.

Variables (*n* = 374)	Category	Frequency	Percent
A diet containing more fruits and vegetables is good to prevent certain diseases	Yes	306	81.8
	No	68	18.2
Know the dietary sources of carbohydrates	Yes	221	59.1
	No	153	40.9
Know the dietary sources of proteins	Yes	178	47.6
	No	196	52.4
Know the dietary source of fat	Yes	107	28.6
	No	267	71.4
Know the dietary sources of fiber	Yes	74	19.8
	No	300	80.2
Know the dietary sources of vitamins	Yes	153	40.9
	No	221	59.1
Know the health consequences of too much consumption of fatty foods	Yes	118	31.6
	No	256	68.4
Know a low intake of fruit and vegetables will cause health problems	Yes	168	44.9
	No	206	55.1
Know the health consequence of much consumption of salt	Yes	221	66.0
	No	127	34.0
Nutritional knowledge	Poor knowledge	211	56.4
	Good knowledge	163	43.6

### 3.4 Types of food groups consumed among adolescent girls in Hurumu Woreda High School

**Figure 1:**
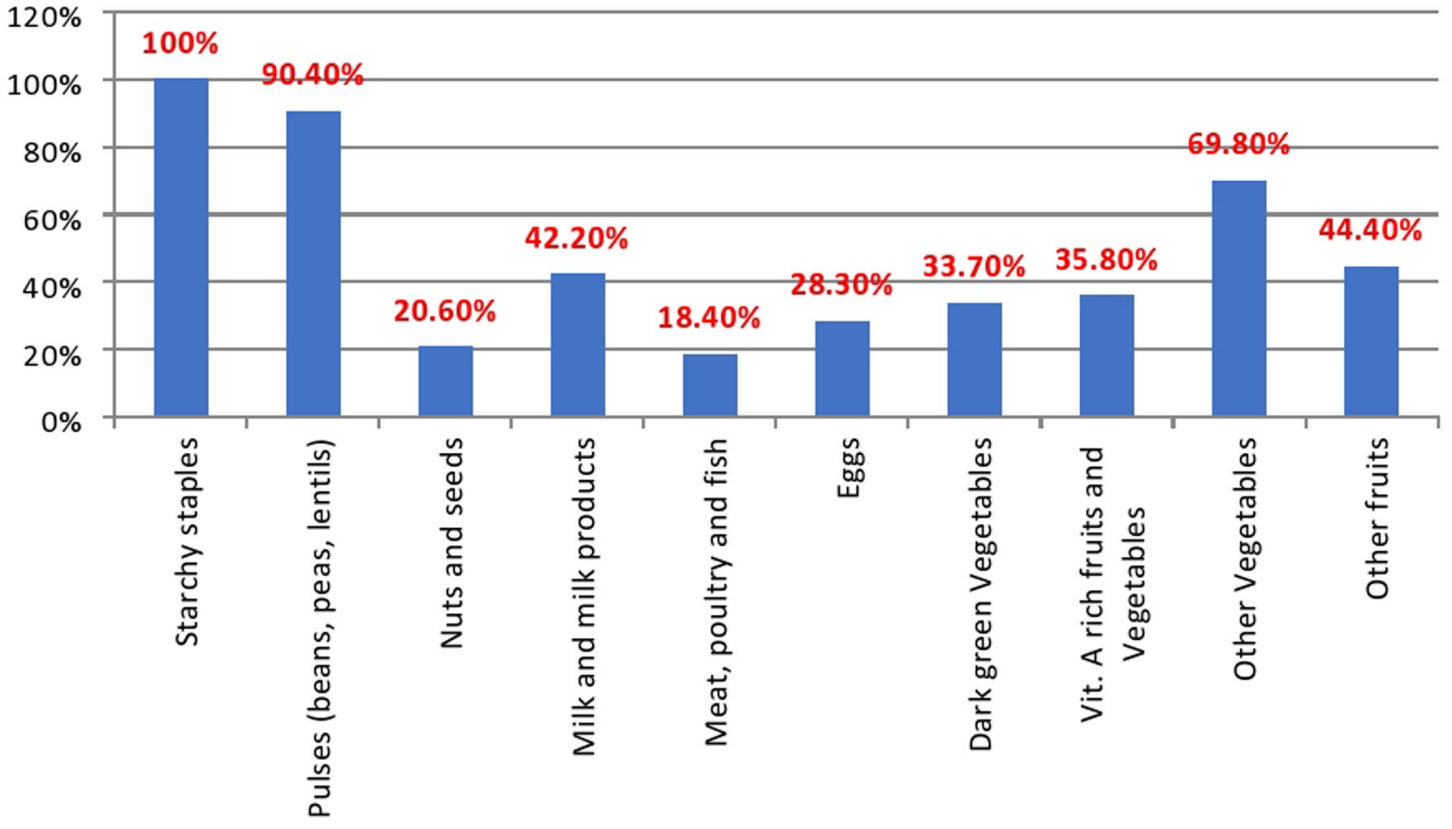
Types of Food groups consumed among adolescent girls in Hurumu Woreda High Schools.

Starch staple foods were the most consumed type of food, accounting for 374 (100%) followed by pulses 338 (90.4%), whereas only 69 (18.4%) of adolescents consumed meat, poultry, and fish during the 24 h (Figure 1).

### 3.5 Dietary diversity score among adolescent girls in Hurumu Woreda High School

The mean dietary diversity score of adolescent girls was 4.37 (SD ± 1.38). The magnitude of inadequate dietary diversity among adolescents in the high schools was 234 **(62.6%)** [95% CI: 57.5–66.5], while 140 (37.4%) of them had adequate dietary diversity.

### 3.6 Multivariable logistic regression analysis of factors associated with inadequate dietary diversity among adolescent girls in Hurumu Woreda High School

On multivariable logistic regression analysis, living with more than five family members (AOR = 1.8 95% CI: 1.16–3.44), consumption of sweet food/beverages (AOR = 2.2 95% CI: 1.07–3.41), poor national knowledge (AOR = 2.5 95% CI: 1.48–3.89), and poor household wealth tercile (AOR = 2.8 95% CI: 1.44–5.12) were all significantly associated with inadequate dietary diversity (Table 4).

**Table 4:** Multivariable logistic regression analysis of factors associated with inadequate dietary diversity among adolescent girls in Hurumu Woreda High Schools, Oromia Region, Southwest Ethiopia, 2022.

Variable (*n* = 374)	Dietary diversity score				*p*-value
	Inadequate	Adequate	COR (95%CI)	AOR (95%CI)	
**Family size**					
<5 members	38 (47.5%)	42 (52.5%)	1	1	
>5 members	196 (66.7%)	98 (33.3%)	2.2 (1.33–3.64)	1.8 (1.16–3.44)*	0.012
**Age of the student**					
14–16	53 (48.2%)	57 (51.8%)	2.4 (1.48–3.69)	1.13 (0.78–2.26)	0.201
17–19	181 (68.6%)	83 (31.4%)	1	1	
**Meal frequency per day**					
Two times	133 (70.7%)	55 (29.3%)	1.5 (1.12–2.14)	1.5 (0.95–2.44)	0.314
3–4 times	121 (58.7%)	85 (41.3%)	1	1	
**Consumption of sweet foods and beverages**					
Yes	85 (79.4%)	22 (20.6%)	3.0 (1.80–5.18)	2.2 (1.07–3.41)*	0.004
No	149 (55.8%)	118 (44.2%)	1	1	
**Nutritional knowledge**					
Poor knowledge	155 (73.5%)	56 (26.5%)	2.9 (1.90–4.53)	2.5 (1.48–3.89)*	
Good knowledge	79 (48.5%)	84 (51.5%)	1	1	0.001
**Occupational status of mother**					
Employed	26 (43.3%)	34 (56.7%)	1	1	
Un employed	208 (66.2%)	106 (33.8%)	2.5 (1.45–4.50)	1.6 (0.43–2.33)	0.093
**Household decision on food service**					
Father	145 (58.2%)	104 (41.8%)	1	1	
Mother	49 (64.5%)	27 (35.5%)	1.3 (0.76–2.21)	1.1 (0.67–1.59)	
Both father and mother	40 (81.6%)	9 (18.4%)	3.0 (1.43–6.85)	1.7 (0.73–4.02)	0.341
**Household wealth status**					
Poor	89 (78.0%)	25 (22.0%)	3.8 (2.23–6.76)	2.8 (1.44–5.12)*	
Medium	79 (64.8%)	43 (35.2%)	2.0 (1.21–3.30)	1.7 (0.97–3.11)	0.002
Rich	66 (47.8%)	72 (52.2%)	1	1	0.061

* denote statistically significant variables in multivariable logistic regression at *p*-value < 0.05, COR, Crude Odds Ratio; AOR, Adjusted Odds Ratio; 1 = reference variable.

## 4 Discussion

Inadequate dietary intake among adolescents can result in increased susceptibility to illness, diminished academic performance, and lower productivity. The long-term implications of insufficient dietary intake during adolescence are significant, as they can have lasting effects on an individual’s physical and mental development, as well as their future potential. This affects not only the individual and their offspring but also the country’s long-term growth potential (9, 23).

This study has attempted to assess the magnitude of inadequate dietary diversity and associated factors among school adolescent girls in Hurumu Woreda. Accordingly, the study found that 62.6% of school adolescent girls in the study area were getting inadequate dietary diversity with mean DDS 4.37 ± 1.38 SD.

In this study, the proportion of inadequate dietary diversity score among school adolescent girls was consistent with a study conducted among school adolescent in Jimma town (61.3%) (12). However, this finding is lower compared with studies conducted in southern Ethiopia (72.4%) (8) and Jimma zone (80.5%) (24). Thus, the inconsistency might be due to a difference in the study period, a difference in the classification of the dietary diversity score, a difference in the study area, or a difference in the number of food groups included. However, this finding is higher compared with study conducted in Tigray, northern Ethiopia (54%) (25), Gondar town (24.6%) (26), Dambi Distirict, northern Ethiopia (32.3%) (20), Adama town (41.2%) (27), Awash town Afar Region (49%) (28), Addis Ababa (43.3%) (13), and Bangladesh (42.3%) (29). The inconsistency might be due to differences in socioeconomic characteristics among the study population and the study period.

In the current study, the mean score of dietary diversity was consistent with studies conducted in Adama town (4.2 ± 2.01) (27), Addis Ababa (4.9 ± 1.47) (13), Woldia, northern Ethiopia (4.73 ± 1.186) (14), Sodo southern Ethiopia (3.56 ± 1.2) (8), and Tigray, northern Ethiopia 3.5 (30), Bangladesh (4.28 ± 1.2) (25), and Pakistan (3.35 ± 1.03) (31). The mean score in this study was higher compared with study conducted in Awash town, Afar region (2.81 ± 0.501) (28) but lower than studies conducted in Tehran (6.25 ± 1.08) (32) and Iran (6.81 ± 1.75) (33). This disparity could be attributed to socioeconomic differences and the presence of food-based dietary guidelines in some countries that encourage the consumption of a diverse diet.

In this study, household wealth status was significantly associated with an inadequate dietary diversity score. Adolescent girls living in households with poor wealth index terciles were 2.8 times more likely to have inadequate dietary diversity compared with those from middle and rich terciles. This finding is consistent with studies conducted in Addis Abeba (34), Meskan District of southern Ethiopia (35), Sodo southern Ethiopia (8), Eastern Uganda (11), Nigeria (36), Bangladesh (10), and Pakistan (31). This implies that living in poverty reduces family food purchasing power, resulting in less access to a diverse and healthy diet (37).

Compared with adolescents living with family size less than five, those living with families greater than five were 1.8 times more likely to have inadequate dietary diversity. This finding is consistent with the study conducted in Woldia northeast Ethiopia (14), and Welfare Monitoring Survey of Ethiopia, 2011 (38). It is an inevitable reality that as the number of family members increases, it becomes more difficult for them to provide adequate and diverse food to meet everyone’s needs (39). As a result, the parent prioritizes and focuses solely on meeting daily needs rather than dietary quality.

Consuming sweet foods/ beverages was found to be significantly associated with the dietary diversity score. Adolescent girls who consume sweet foods/drinks were 2.2 times more likely to have inadequate dietary diversity compared with those who do not consume sweet foods/beverages. This finding was consistent with a study conducted in Addis Ababa, which reported that dietary diversity was negatively associated with the consumption of sugar-sweetened soft drinks (13). This could be because sweet foods or sugar-sweetened soft drinks raise blood sugar levels, resulting in a loss of appetite and a decrease in the variety of food intake (40, 41).

Nutritional knowledge was significantly associated with the dietary diversity score. Adolescents with a poor knowledge of nutrition were 2.5 more likely to have inadequate dietary diversity compared with those who have good knowledge of nutrition. This finding was consistent with studies conducted in Luxembourg (42), Mandera County, Kenya (43), and Woldia northeast Ethiopia (14). This implies that adolescents with poor nutritional knowledge are less likely to consume nutritious and diverse foods. This finding is supported by a study conducted in a Taiwanese elementary school, which found that students with good nutritional knowledge were more likely to have a high dietary diversity score (44).

### 4.1 Limitation of the study

This study has its own limitations. First, the study assessed individual dietary diversity only for the last 24 h. Hence, there might be a lack of a correct reflection of the usual dietary habits. Second, there might be recall biases and social disability biases. These limitations were reduced by giving training on techniques for reducing recall biases and social desirability biases. Third, due to time and cost constraints, this study did not address portion size estimation of food consumed by participants.

## 5 Conclusion and recommendation

In this study, more than half of adolescent girls had inadequate dietary diversity. Living with more than five family members, poor wealth status, eating sweet foods or beverages, and poor nutritional knowledge were factors significantly associated with an inadequate dietary diversity score. Therefore, designing a nutrition education at schools and utilization of family planning methods to establish a small family size will help to achieve the consumption of adequate dietary diversity. Furthermore, income-generating activities should be implemented to improve the household’s economic status, which will increase their purchasing power for a variety of foods.

## Data availability statement

The raw data supporting the conclusions of this article will be made available by the authors, without undue reservation.

## Ethics statement

Ethical clearance was obtained from the ethical review board of Mattu University, College of Health Science (Ref. No. RPG 71/22) submitted to Hurumu Woreda education office. The letter of permission was obtained from Hurumu Woreda education office. The nature of the study was fully explained to the study participants and parents/guardians. Informed verbal and written consent was obtained from parents/guardians for adolescent girls aged less than 18 years old and assent was obtained from the participant before the interview.

## Author contributions

AK developed the research idea, design, analysis, and drafted the manuscript. AZ, KM, ZB, and TB conceived the study, supervised the data collectors, interpret the result, and reviewed the manuscript. AK is responsible for the overall content as guarantor. All authors contributed to the article and approved the submitted version.
